# Anime watching in childhood may affect suicidal risk factors in adult life

**DOI:** 10.1192/j.eurpsy.2024.1628

**Published:** 2024-08-27

**Authors:** M. Marachev, V. Rudchenko, A. Grigorieva, L. Usova, A. Mokritskaya

**Affiliations:** ^1^ Moscow Scientific Research Institute of Psychiatry Branch of V. Serbsky National Medical Research Centre for Psychiatry and Narcology; ^2^ Neurocenter of medical and psychological correction and rehabilitation; ^3^I.M. Sechenov First Moscow State Medical University (Sechenov University), Moscow; ^4^Ryazan State Medical University named after academician I.P. Pavlov, Ryazan; ^5^I.M. Sechenov First Moscow State Medical University (Sechenov University), Moscow, Russian Federation

## Abstract

**Introduction:**

Suicide is one of the leading causes of death worldwide being the fourth major cause of death among young people 15-29 years old. The reduction of suicide mortality is prioritized by the World Health Organization (WHO, 2019). There is a number of internal and external factors associated with suicidality (Soto-Sanz V et al., 2019; Farbstein et al., 2022.). Special attention is paid to the influence of the social media on suicidality (Cheng A. T. A. et al., 2007; Niederkrotenthaler T. et al., 2020; Sedgwick R. et al., 2019). In the Russian Federation, anime, an animation genre and a media cultural phenomenon, is increasingly popular among young people. Characters who are lonely and lost their meaning of life are common in anime. Romanticization and idealization of such characters may lead to increased attractiveness of death and thus have a negative effect on the mental health of adolescents and young adults due to their incomplete identity development (Liu Y. et al., 2022; Backer, H. A., 2023).

**Objectives:**

We aimed to study the influence of the anime on the presence of suicidality and depression in adolescents and young adults in the Russian Federation.

**Methods:**

We interviewed 304 people living in the Russian Federation and watching anime on the regular basis (244 women, mean age 20.9 ± 3.8 years, range 13-36 years). We collected sociodemographic data and age when a person had started watching anime. We performed Reasons for Living Inventory, RFL (M. Linehan et al., 1983), Beck Depression Inventory, BDI (Aaron Beck, 1961). We divided all participants into three groups according to their age: adolescents (13-19 years), young people (20-24 years), adults (25-36 years). In each group, we compared BDI: level of depressive symptoms, cognitive-affective subscale, subscale of somatic manifestations of depression; RFL scales: Survival coping beliefs, responsibility to family, child related concerns, fear of suicide, fear of social disapproval, moral objections between three subgroups based on the age of the anime watching start (<12 years old, 12-15 years old, ≥16 years old) using Kruskall-Wallis test and post hoc Mann-Whitney U-test for pair comparisons with Bonferroni correction for multiple comparisons. Level of significance p<0.05.

**Results:**

In the adolescents (n=130), we did not find any differences between the three subgroups. In the young people (n=127), participants who had started watching anime in childhood (<12 years old) had higher level of depression (p= 0,014) and higher level of cognitive-affective symptoms (p= 0,006). In the adults (n=47), participants who had started watching anime in childhood had decreased moral attitudes contrary to suicide (p= 0,004). Other scales not found to differ significantly.

**Conclusions:**

Start of the anime watching in childhood (<12 years old) was associated with increased suicidal risk factors and decreased anti-suicidal factors in the young adults.

**Disclosure of Interest:**

None Declared

